# The State Medical Association

**Published:** 1901-05

**Authors:** 


### TEXAS MEDICAL JOURNAL.

AUSTIN, TEXAS.

A MONTHLY JOURNAL OF MEDICINE AND SURGERY.

EDITED AND PUBLISHED BY
F. E. DANIEL, M. D.

ASSOCIATE EDITOR:
WITTEN BOOTH RUSS, M. D.,
San Antonio, Texas.

Published Monthly at Austin, Texas. Subscription price $1.00 a year in advance.

Eastern Representative: John Guy Monihan, St. Paul Building, 220 Broadway, New York City.

Official organ of the State Association of Health Officers, the West Texas Medical Association, the Houston District Medical Association, the Austin District Medical Society, the Brazos Valley Medical Association, the Galveston County Medical Society, and several others.

### The State Medical Association.

At the thirty-third annual meeting of the Texas State Medical Association just closed, according to the report of the secretary, published herewith, the roll showed an active membership of 297. Eighty-one new members were admitted—swelling the aggregate to 378. This is not a very creditable showing after thirty-three years of earnest endeavors to organize the medical profession of Texas. It demonstrates that something is wrong with the methods or policy of the Association, and demands that a change in those methods should be made. Just where the fault lies, I do not know, but it is my deliberate opinion that the policy always pursued of flying from one extreme of the State to another to hold the annual meetings, is the principal factor in the disintegration that is always going on, the lack of cohesion. The membership is not fixed, but ever shifting. There is a condition existing analogous to *metabolism,* a building up and a waste, or tearing down, constantly going on,—and a kind of equlibrium is established, which maintains the membership at somewhere between three hundred and four hundred. There is no lack of new members; they come in by the score at nearly every meeting,—but as the Association for the next two or three years thereafter will meet at some distant point out of convenient reach of the rank and file—the country doctors mostly (and they are the "salt of the earth"—the backbone of the profession)—a corresponding number is dropped from the rolls annually for non-payment of dues. These men are thus deprived of the benefit and the pleasure of the meetings, and they fail to keep up their membership.

This has been going on for many years, and the roll shows a net gain of only ninety-eight in the seventeen years since April, 1884, and an actual loss of eight members since April, 1894.

From 1884 to 1894, inclusive, five hundred and twenty-one new members were admitted, which, added to the 280 on the rolls in 1884, swelled the aggregate to 801. In the same decade four hundred and fifteen were dropped, leaving the membership 386 in 1894, just about where it is now. Since 1894 a very large accession has been made at each meeting, yet the roll today shows a loss of eight since '94.

In my judgment the decision to hold the 1902 meeting at El Paso was a serious mistake. It was not well considered. It is not for the best interest of the Association—nor that of the profession at large. It will deprive the great majority of the members of the privilege of attending. It is out of their reach, and is a rank injustice to the boys from the forks of the creek.

Personally, I have no objections. It suits me. I want to see El Paso, and while personally I do not like to contemplate a journey of eighteen hundred miles—across a desert for the most part, nearly nine hundred miles each way to and from Austin, and requiring in all about three days and two nights, or three nights and two days—I shall attend—*D. V.;*—and so will the railroad surgeons, and the college professors, and the rich specialist, and others who can afford it,—but the average country doctor cannot afford to go so far, nor be gone so long from home, nor to pay even "one fare and ten per cent. round rate,"—the best rate we have ever been able to get.

Let me see: Say from the Capitol, Austin, to Fort Worth, 200 miles; Fort Worth to El Paso, 650 miles—850 miles at three cents per mile, $25.50, ten per cent. added, $2.55, $28.05. Sleepers four nights (going and coming) $8.00. Hotel three and one-half days, $10.50;—makes a total cost of $56.55, not including meals on train and other incidentals—and an absence from home of about eight days. The trip from Austin via San Antonio might be a little less expensive; but the trip by either route will be more expensive to those living in the remoter parts of the State.

It is said by those who insisted upon meeting at El Paso—the extreme northwestern part of this great, wide empire, 750x850 miles in extent—that they "will try to get a $10 round trip rate." I do not believe it can be done. Let my readers figure on the cost —each one for himself—to go to El Paso even at a $10 rate. I much fear that the meeting will be numerically a failure and a big hole will be thereby made in the treasury pile, which the handsome and courteous young treasurer, Dr. Miller, has collected.

It was my intention to oppose the selection of El Paso when the nominating committee should make its report; but they made the report Thursday p. m. instead of Friday morning, *as required by the constitution,* at a time when those who, like myself, objected to such an unwise course, expected them to make it,—and hence we were given no opportunity to protest. Not for myself, by any means, do I protest,—but in the interest of the members of the profession who cannot afford to attend. I intended to make the fight for the "boys," because they are being deprived—through no fault of theirs—of the benefits of the meeting, for which they annually pay their hard-earned dollars. I say,—in my opinion, the policy of skipping from Tyler, Sherman or Paris to Houston or Galveston,—from Galveston to El Paso—perhaps to Dallas,—a sort of "now you see me, now you don't"—should be abandoned. It is unwise—unjust to the rank and file—the *majority,* ruinous to the Association, and is the principal cause of the crumbling away of the membership, despite the large accessions every year. There are no physicians (except in El Paso) within four hundred miles of El Paso. I fear there will not be fifty members present, and we cannot expect many additions to the membership, but the decision has been made. El Paso it is, and we shall see what we shall see. It is a *mistake.* It should be reconsidered still, and it can be done. Change it to Dallas. I fear the better judgment of the nominating committee was in abeyance—they were hypnotized by the reiterated assurances of a "good time" and the opportunity to see that wonderful country. That is not just exactly what the average doctor pays his membership fee to do or to get.

Some change in the methods or policy of the Association should be made. We have played a losing game twenty years, let us try something else; say, let us meet every year at Galveston, Houston or San Antonio, or at Dallas or Fort Worth,—points accessible to the largest number of physicians, and not *wait for an invitation.*

